# Supplementary material for: New holostean fishes (Actinopterygii: Neopterygii) from the Middle Triassic of the Monte San Giorgio (Canton Ticino, Switzerland)
Source: PeerJ. 2016 Jul 19;4:e2234. doi: 10.7717/peerj.2234 (PMC4957996; doi:10.7717/peerj.2234)
Supplement: Appendix S1 [file peerj-04-2234-s001.pdf]

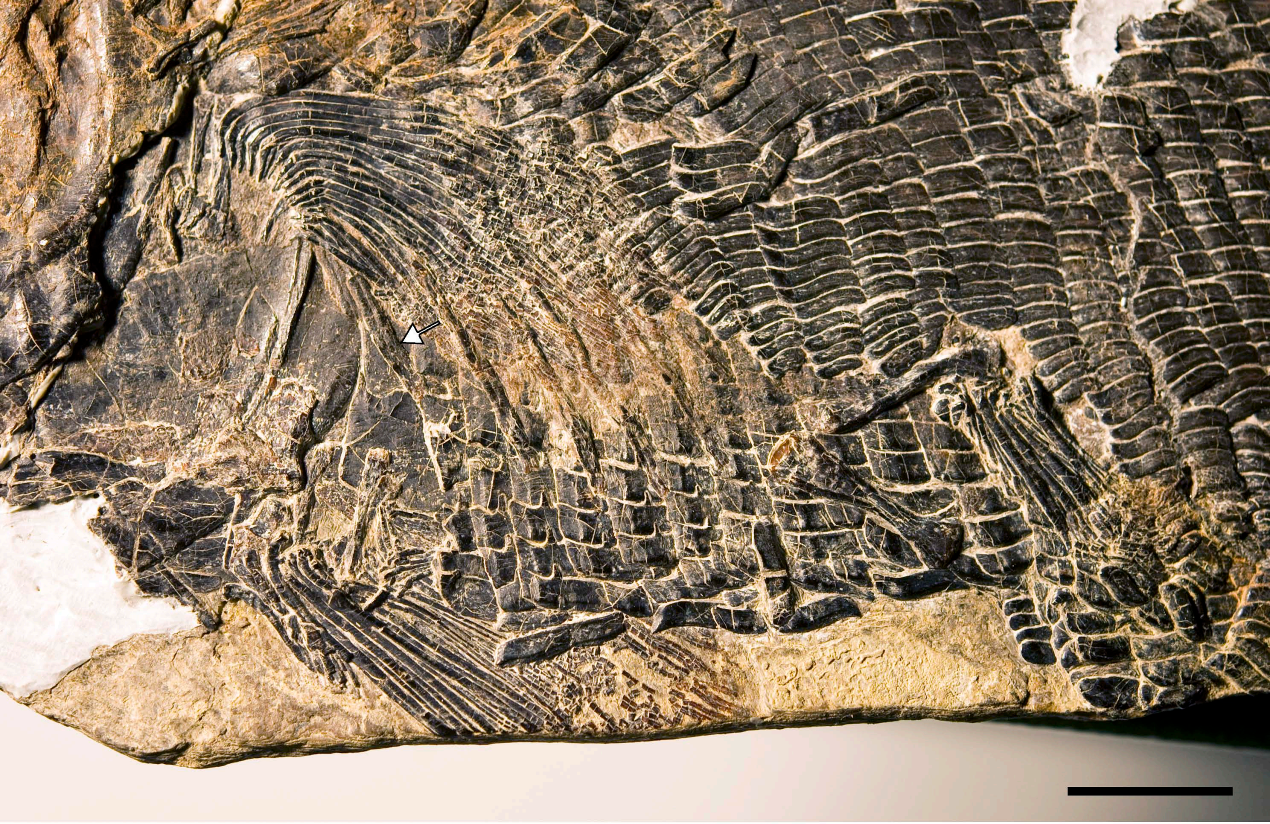

S1-Figure 1. *Ticinolepis longaeva* gen. et sp. nov., photograph of MCSN 8475 preserved in left lateral view showing ribs, pelvic bones and several articulated scales exposed in mesial view. Small arrow point to first preserved rib. Scale bar = 1 cm.

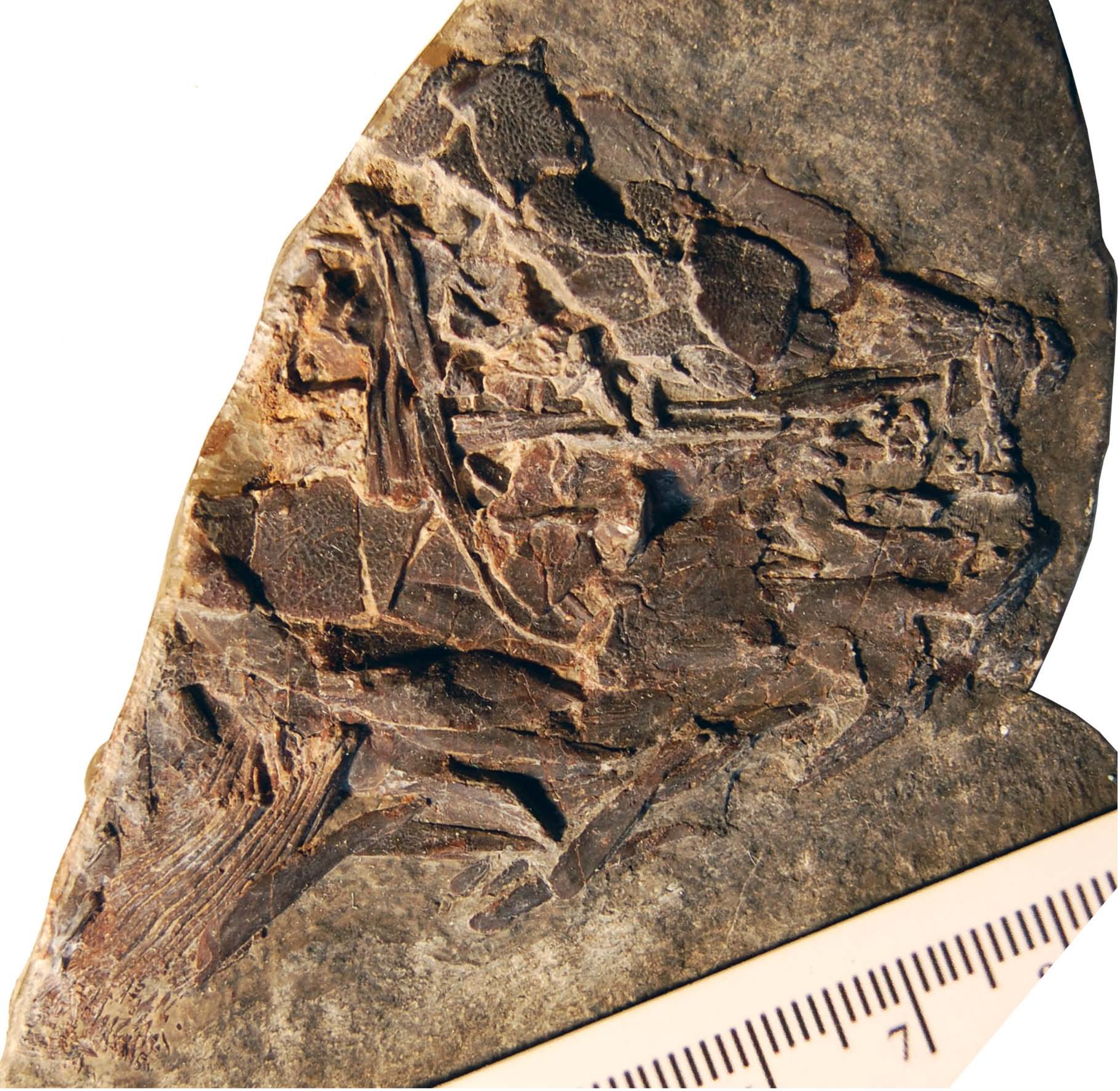

S1-Figure 2. *Ticinolepis longaeva* gen. et sp. nov., photograph of MCSN 8317 preserved in right lateral view showing partially preserved braincase and other skull bones and partially preserved pectoral girdle and fin.

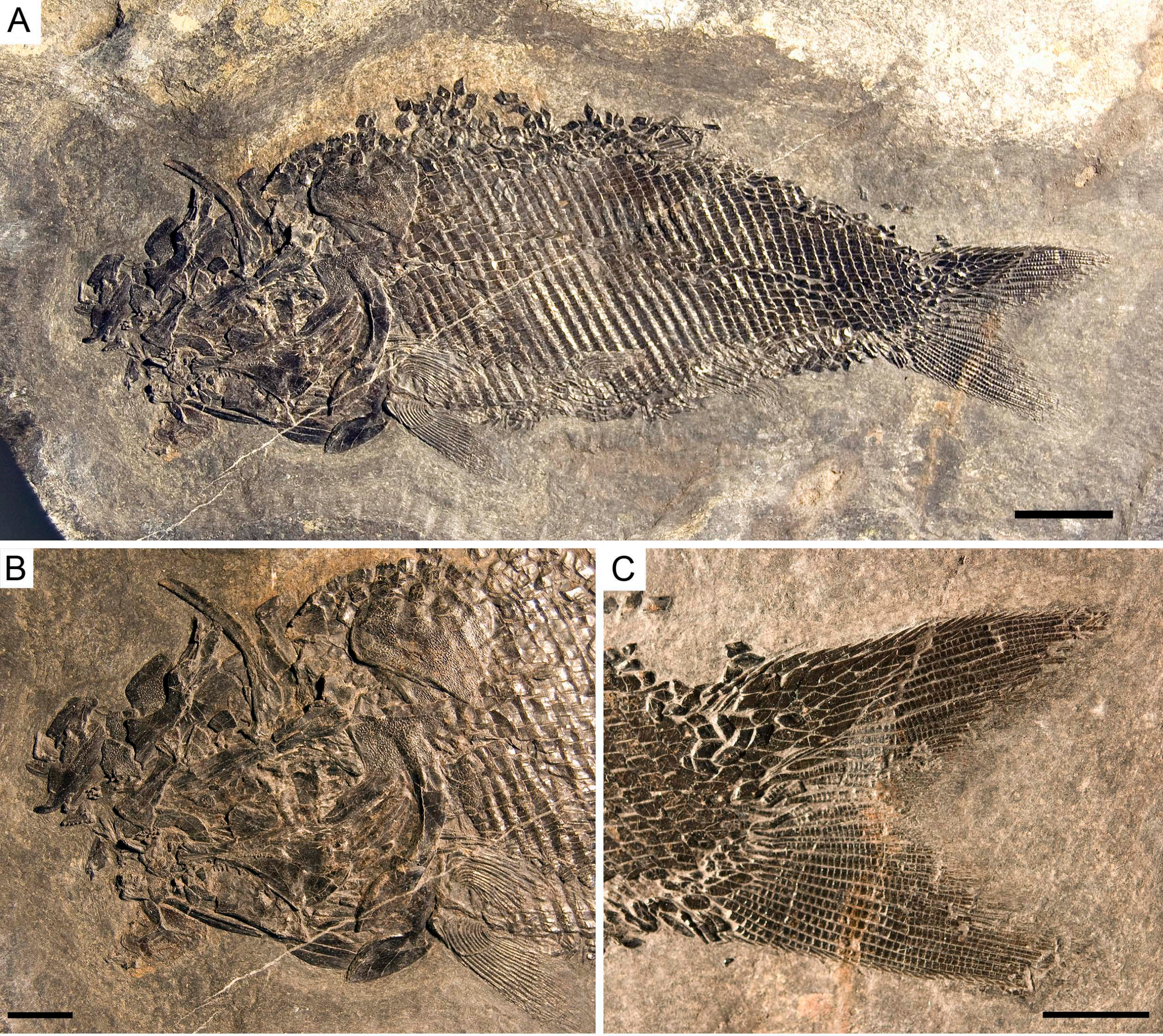

S1-Figure 3. *Ticinolepis longaeva* gen. et sp. nov., photographs of MCSN 8351 preserved in left lateral view. A, Overview of the complete specimen; scale bar = 2 cm. B, Skull; scale bar = 1 cm. C, Caudal fin; scale bar = 1 cm.

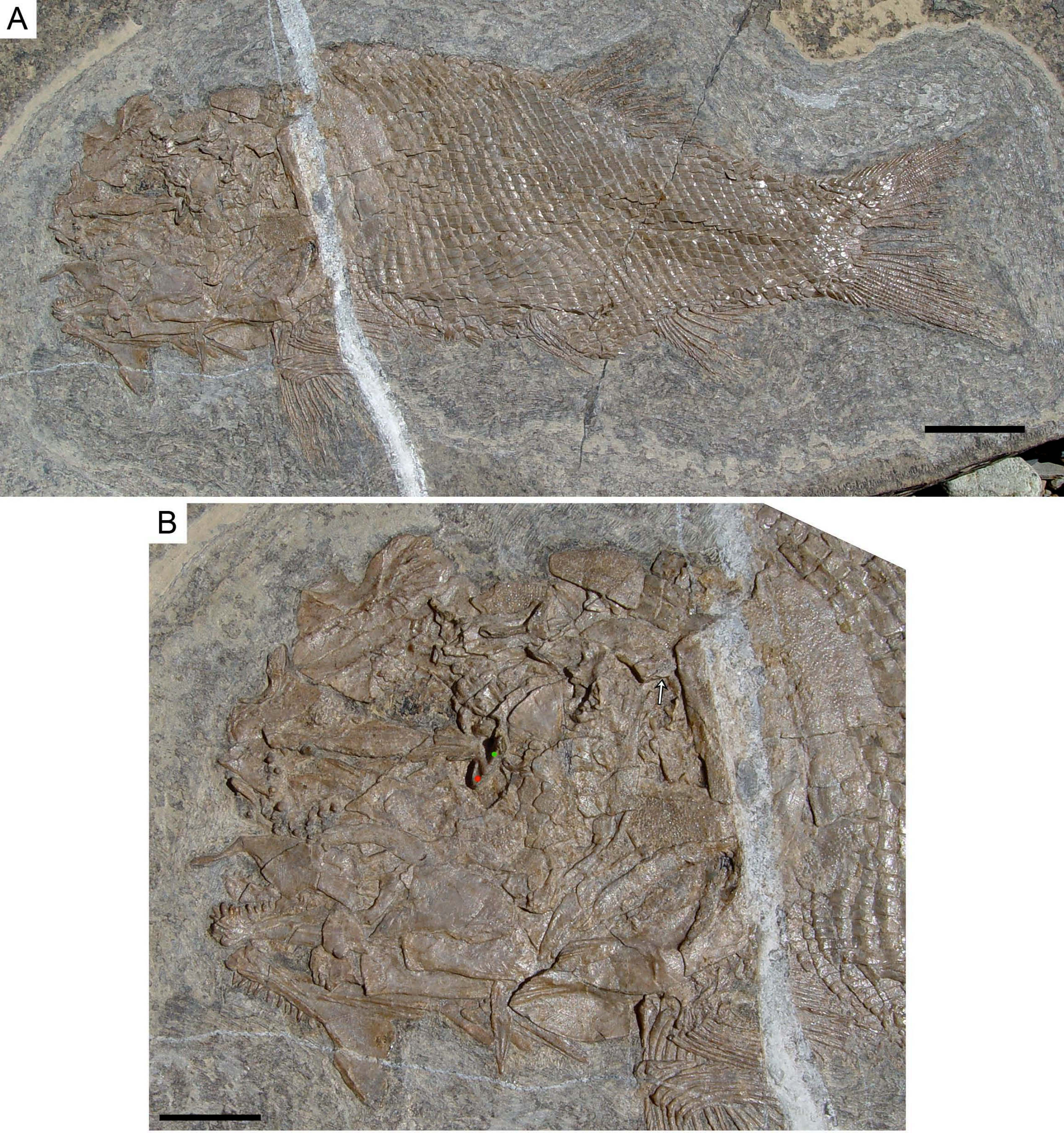

S1-Figure 4. *Ticinolepis longaeva* gen. et sp. nov., photographs of MCSN 8008 preserved in left lateral view. A, Overview of the complete specimen; scale bar = 2 cm. B, Skull; scale bar = 1 cm.

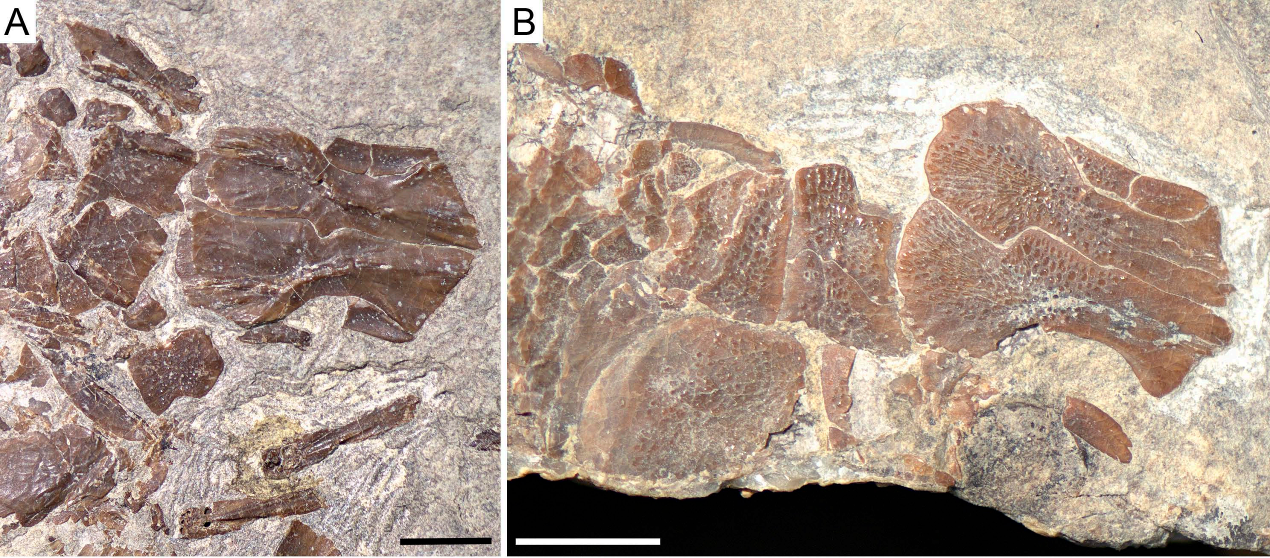

S1-Figure 5. *Ticinolepis longaeva* gen. et sp. nov.. A, Disarticulated skull of PIMUZ T 4785 preserved mostly in mesial view. B, Partially articulated skull of PIMUZ T 4788 preserved mostly in right lateral view with the detached frontals and left supraorbitals preserved in dorsal view. Scale bars = 0,5 cm.

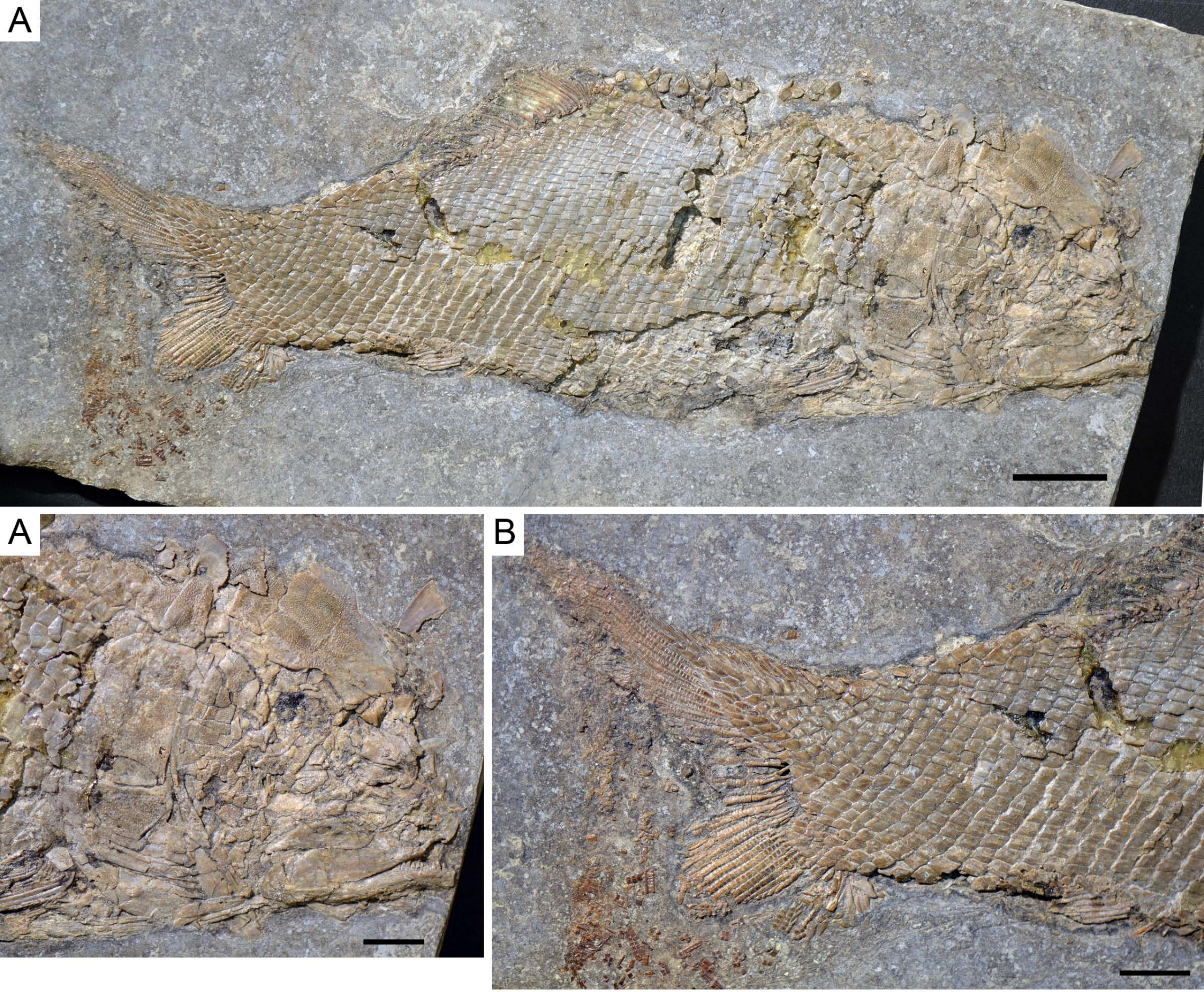

S1-Figure 6. *Ticinolepis longaeva* gen. et sp. nov., skull of PIMUZ T 4987 preserved in right lateral view. A, Overview of the complete specimen; scale bar = 2 cm. B, Skull; scale bar = 1 cm. C, Caudal fin; scale bar = 1 cm.

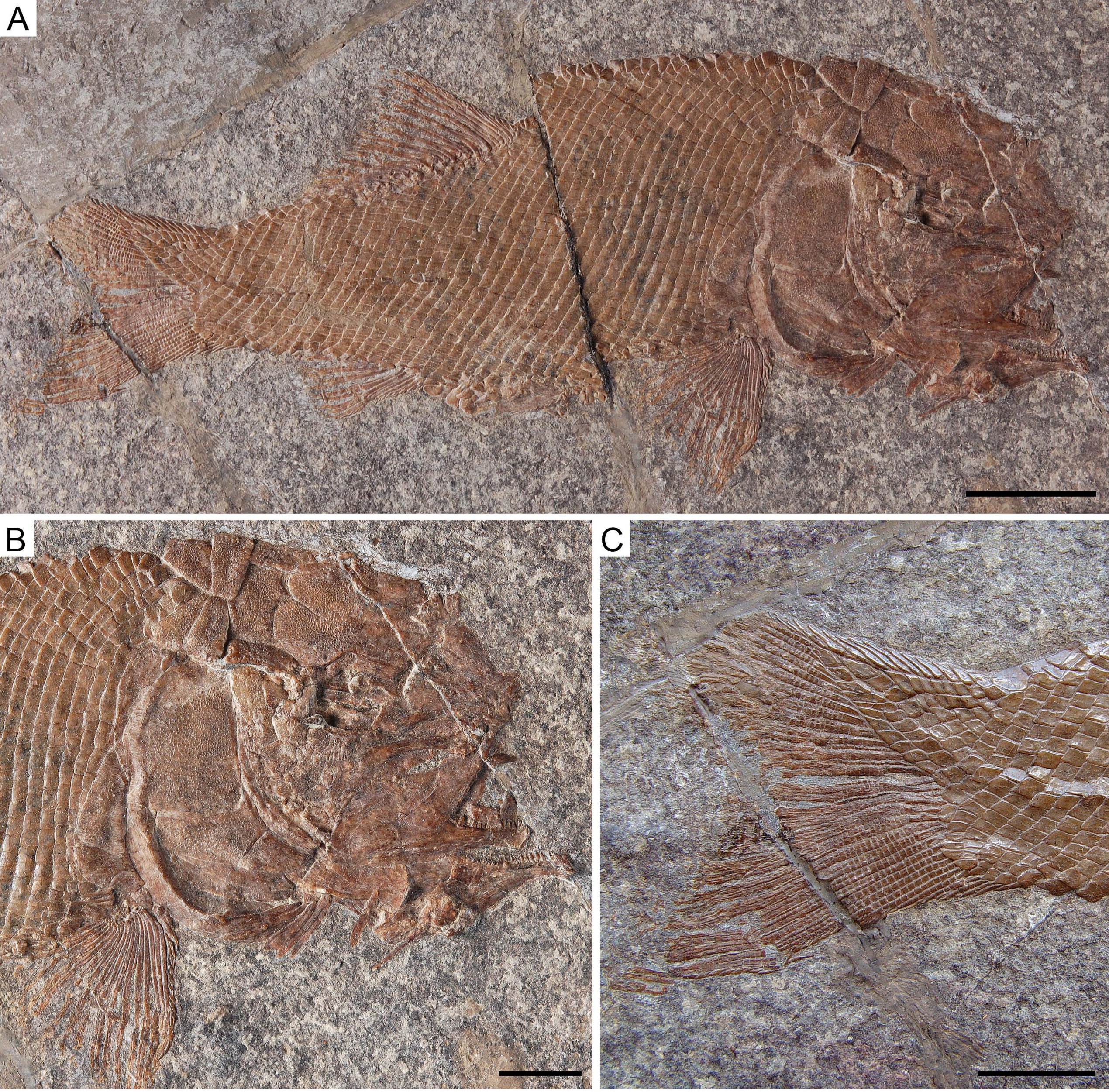

S1-Figure 7. *Ticinolepis longaeva* gen. et sp. nov., skull of MCSN 8073 preserved in right lateral view. A, Overview of the complete specimen; scale bar = 2 cm. B, Skull; scale bar = 1 cm. C, Caudal fin; scale bar = 1 cm.

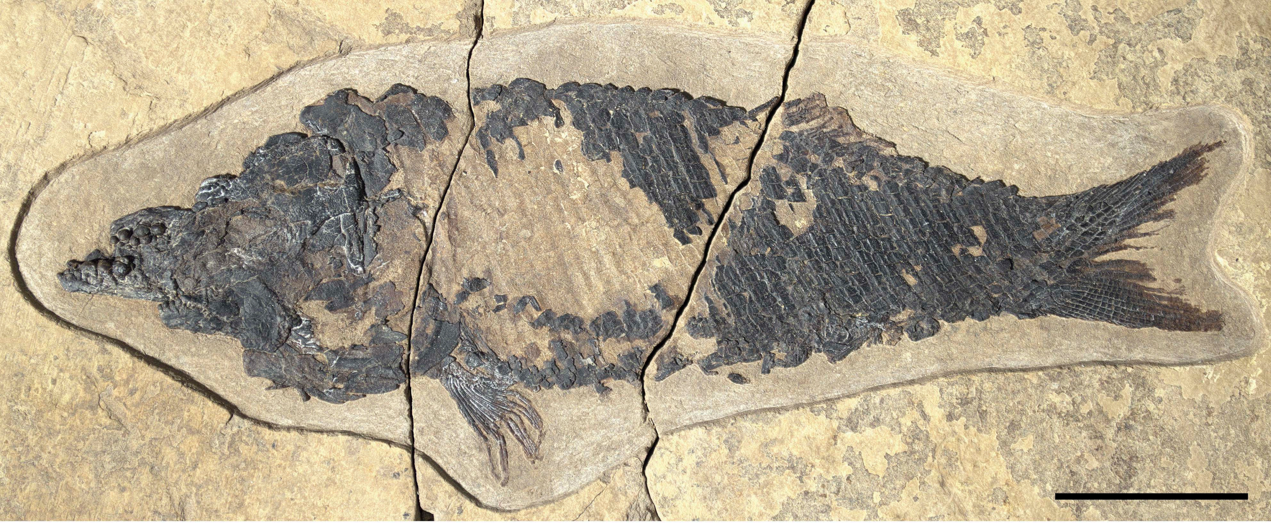

S1-Figure 8. *Ticinolepis crassidens* sp. nov., PIMUZ T 438 preserved in left lateral view except for the skull roof, which is disarticulated and preserved in dorsal view. Overview of the complete specimen; scale bar = 2 cm.

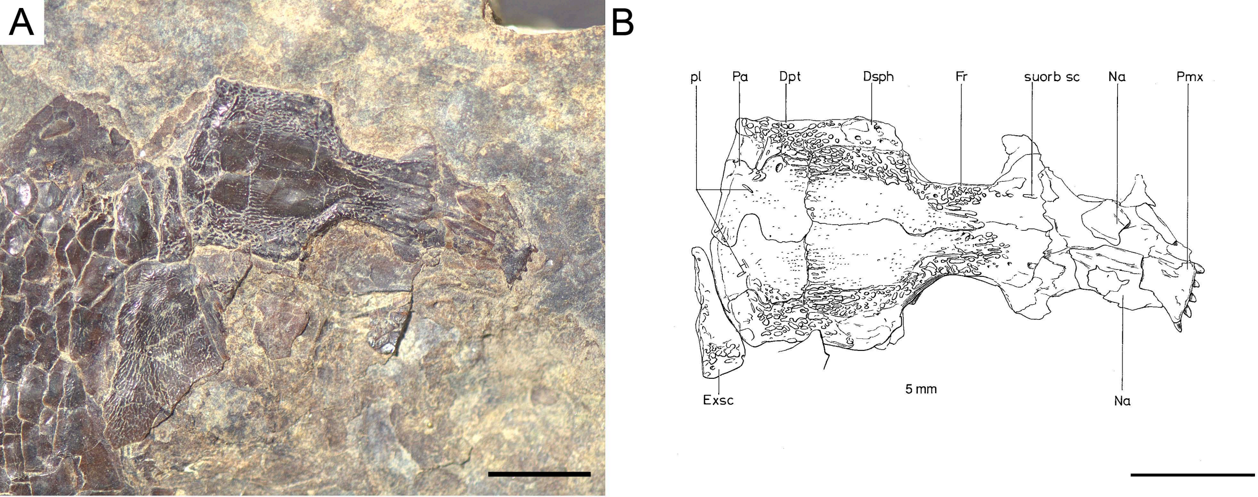

S1-Figure 9. *Ticinolepis crassidens* sp. nov., skull roof in PIMUZ T 401 preserved in dorsal view. A, Photograph. B, Line drawing. Scale bars = 5 mm.

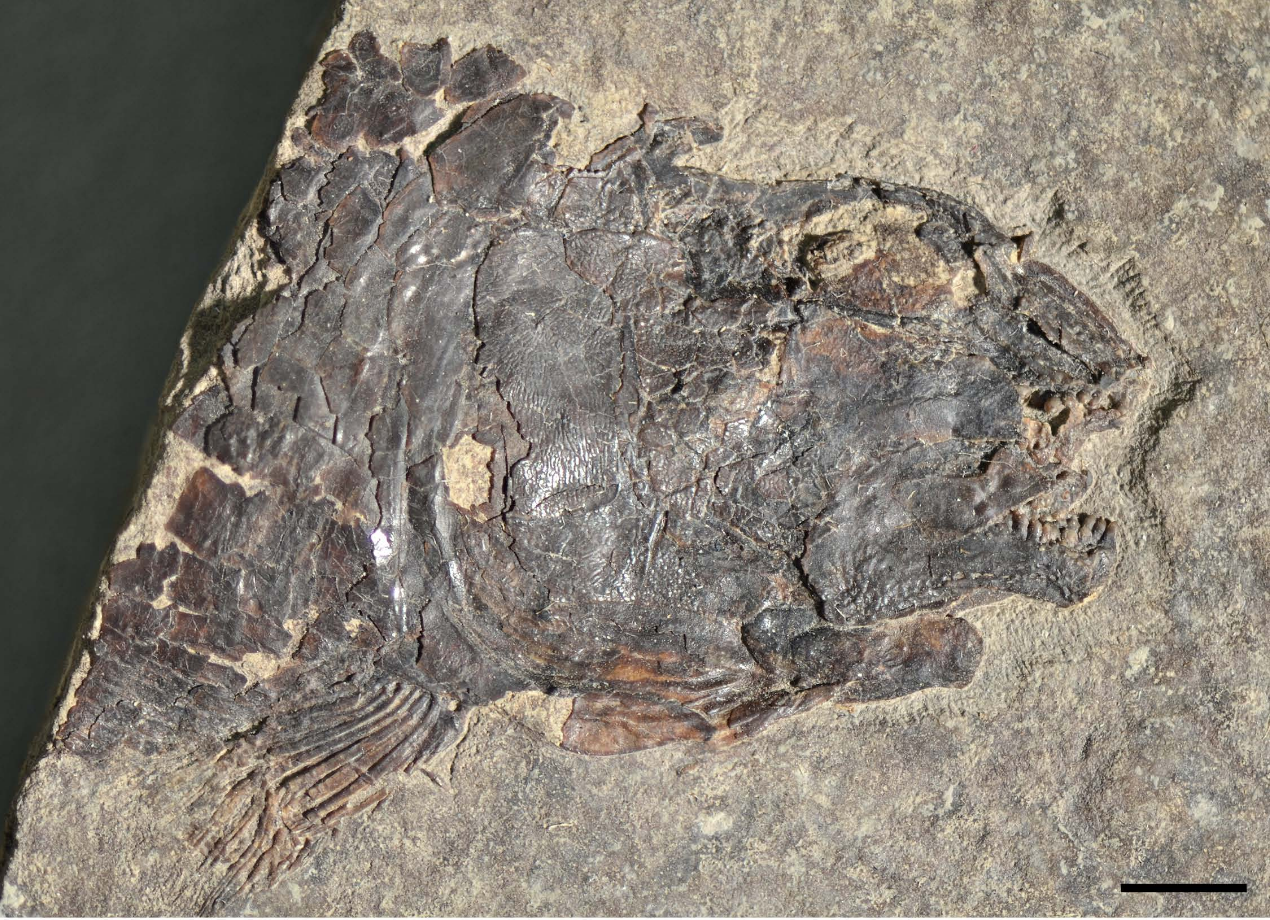

S1-Figure 10. *Ticinolepis crassidens* sp. nov., skull in PIMUZ T 2823 preserved in right lateral view. Scale bar = 5 mm.
